# Effects of Early Sport Specialization on Injury Load Management and Athletic Success of National Basketball Association Players

**DOI:** 10.1177/23259671241304732

**Published:** 2025-01-03

**Authors:** Luke Sang, Katherine Bach, Brian T. Feeley, Nirav K. Pandya

**Affiliations:** * School of Medicine, University of California, San Francisco, San Francisco, California, USA; † Department of Orthopaedic Surgery, University of California, San Francisco, San Francisco, California, USA; Investigation performed at the Department of Orthopaedic Surgery, University of California, San Francisco, San Francisco, California, USA

**Keywords:** pediatric sports medicine, basketball, injury prevention, sport specialization

## Abstract

**Background:**

The effects of early sport specialization on professional athletes’ resilience in handling increased workloads and athletic success have not been fully described.

**Hypothesis:**

National Basketball Association (NBA) players who were multisport athletes during high school would be able to withstand higher workloads with lower injury rates and have more athletic success compared with their single-sport peers.

**Study Design:**

Descriptive epidemiology study.

**Methods:**

Included were first-round NBA draft picks from 2013 to 2023 who had played ≥1 game in their first 3 seasons after being drafted. Athletes who had participated in ≥1 high school sports in addition to basketball were classified as multisport athletes, while those who had only played basketball were classified as single-sport athletes. For each player's first 3 NBA seasons, workload data (number of games played and distance traveled per game/season in meters), injury history, statistical performance (player efficiency rating), and end-of-season award history were collected through the official NBA advanced statistics database and through publicly available records.

**Results:**

Overall, 318 athletes were included, of whom 87 (27.4%) were multisport and 231 (72.6%) were single-sport. During their first 3 seasons combined, multisport athletes played in significantly more games (148.9 ± 67.1 vs 125.8 ± 63.8; *P* < .01), traveled greater total distances (133,183.9 ± 239,923.0 m vs 73,879.5 ± 165,093.9 m; *P* < .01), and had a significantly lower percentage of games missed due to injury (13.5% vs 16.9%; *P* < .001) compared with single-sport athletes. There was a significant correlation between increased workload (total distance traveled) and number of injuries in single-sport athletes (*ρ* = 0.37; *P* < .001) but not in multisport athletes (*ρ* = 0.14; *P* = .20). Last, multisport players had a significantly higher player efficiency rating (12.8 ± 11.6 vs 10.5 ± 5.1; *P* < .05) and award achievement likelihood (40.2% vs 19.0%; *P* < .001).

**Conclusion:**

NBA players who had participated in multiple sports during high school demonstrated an ability to withstand higher workloads while having fewer games missed due to injury when compared with players who had only played basketball. Furthermore, athletes who delayed sport specialization had greater statistical and award success in their professional careers than those who focused on early single-sport specialization.

Youth participation in sports has consistently increased over the past few decades. Based on a participation survey conducted by the National Federation of State High School Associations in 2018-2019, participation was at almost 8 million compared with just 6.7 million in 2000-2001.^41^ Somewhat paradoxically, there has also been a rise in single-sport specialization for youth athletes, and it is now much less common for high school athletes to play multiple sports.^2,3^ Single-sport specialization is thought to be beneficial in reaching an “elite,” or professional, status because of the ability to increase the focus and volume of specific skill training.^4,10,18^ However, these claims are largely unsupported by research, and sport specialization remains controversial despite the rising number of single-sport youth athletes.^29^

High school sports participation has generally been utilized to serve as a strong indicator for early sport specialization^35,39^ because the definition of early sport specialization set by the American Academy of Pediatrics (ie, single-sport participation before the onset of puberty) and the mean age of puberty for the majority of boys (13-14 years) coincides with the mean age of starting high school in the United States.^3,40^ Recent studies have shown that early sport specialization provides no advantage over multisport athletes in reaching elite levels in their sport.^11,14,30^ In fact, single-sport specialization has been linked to increased physiological burnout^19,22^ while also increasing the risk of overuse injury.^5,11,19,31^ Bell et al^2^ demonstrated that highly specialized high school athletes were more likely to report a history of overuse knee injury than multisport athletes. Based on these findings, multiple groups, including the American Academy of Pediatrics, have generally advocated for and recommended delayed sport specialization.^3,16,26,33^

Even after reaching an elite level, single-sport athletes have been shown to have shorter careers and more injuries compared with athletes who participated in multiple sports as adolescents.^6,35,39^ In a study of athletes in the National Basketball Association (NBA), Rugg et al^35^ found that players who were multisport athletes in high school played in more games and had fewer major injuries than single-sport players. However, the specific differences between multisport and single-sport athletes regarding resilience to increased physical workload and achievement of athletic success in team sports have yet to be thoroughly studied. With the recent rise in the NBA of the concept of *load management*—the practice of resting players with the hope of preventing overuse injury—it is crucial to parse out the differences between multisport and single-sport athletes in their ability to handle higher workloads and avoid injury.^32^

The purpose of this study was to compare the differences in workload, both per game and over the course of the entire season, and the rate of injury between NBA players who were multisport athletes in high school and those who only played basketball. Further, we identified differences in athletic success through statistical measures and end-of-season awards between these 2 groups. We hypothesized that NBA players who were multisport athletes in high school would be able to withstand higher workloads with lower rates of injury and would achieve more athletic success compared with their single-sport counterparts.

## Methods

### Study Design and Participants

This was a retrospective review of the official NBA advanced statistics database and publicly available information on NBA first-round draft picks from 2013 to 2023. First-round draft picks for all teams from 2013 to 2023 were identified, and inclusion criteria included playing ≥1 regular season game in their first 3 seasons after being drafted. Only first-round draft picks were included due to their representation of the most “elite” pool of athletes and the increased likelihood of having available performance, injury, and past sporting information due to their increased prominence. From the original pool of 330 players, 12 were excluded due to their lack of games played during their first 3 seasons, resulting in a final sample size of 318 athletes.

We collected a comprehensive set of basketball data on each player's first 3 seasons after being drafted. This data set included the number of games played, distance covered (in meters) per game and over an entire season, player efficacy rating (PER), and end-of-season award achievements. We used data from the first 3 seasons after being drafted to create a standardized sample across all players for their playing careers during which they most likely had not yet suffered any major, performance-altering injuries. For players recently drafted and who had not yet played 3 seasons, all data of their NBA career up to the end of the 2023 calendar year were utilized, and their overall data metrics were adjusted to account for only the number of seasons and games they had participated in. These players were included to both maximize our sample size and incorporate the most recent set of athletes to experience the current early sport specialization pressures and environment. Only regular-season games were considered in this study; both preseason and playoff games were excluded. Workload data, such as games played and distance traveled per game and over an entire season, were acquired using the official NBA advanced statistics database (www.nba.com/stats). PER and end-of-season award achievements were gathered from www.basketball-reference.com. End-of-season award achievement was defined as a player's being selected to an All-Rookie or All-NBA team at least once in his first 3 seasons after being drafted.

We gathered injury information for each player's first 3 seasons, including the number of injuries, injury descriptions, and games missed due to the injury. An injury was included in the data set if it affected the neck, shoulders, torso, back, hips, groin, legs, and feet. Injuries such as upper extremity fractures, concussions, and facial fractures were excluded, as these injuries are less likely to represent overuse-related injury in basketball. The injury must have occurred during a basketball-related activity at the professional level to be counted toward the data set. All injuries caused by other events, such as a motor vehicle accident, were excluded. Injury that caused >10 games to be missed was additionally noted to be a major injury. Injury information was collected through standard internet web searches, as previously described in other studies on professional athletes.^8,27,28,35^ Representative common search patterns are detailed in Appendix Table A1. Examples of the websites utilized included www.espn.com, www.foxsports.com, www.cbssports.com, www.si.com, www.nba.com, and www.sports.yahoo.com.

Utilizing publicly available sources on the internet, we gathered information for each player for single-sport or multisport classification based on his high school sporting history.^3,35,39,40^ Thus, to be classified as a multisport athlete, the player must have had evidence of participation in a sport other than basketball during high school. Participation in other sports that ended before high school, such as during middle school, was not counted toward multisport classification. For foreign players, who might not have the same structure of high school sports in their countries, multisport participation was analyzed in the typical age range of US high school athletes (13-18 years).^2^ We used searches through all publicly available information, including but not limited to player interviews, biographies, newspaper articles, and high school sports services (ie, www.maxpreps.com, www.athletic.net).

### Statistical Analysis

Unpaired *t* tests were used to determine significant differences in workload (games played, total distance traveled, distance per game), injury, and PER between single-sport and multisport athletes across individual seasons and all first 3 seasons. Two-tailed chi-square tests with Yates correction were utilized to determine significant differences in aggregate games missed due to injury and end-of-season award achievements between single-sport and multisport players. Yates correction was used to prevent overestimation from unequal cohort sizes and for a more conservative statistical analysis. Spearman correlation analysis was performed to determine the association between player workload and injury in the single-sport and multisport cohorts, individually and combined. Statistical significance was considered at *P* < .05. All analyses were performed using Stata 18 (StataCorp LLC).

## Results

A total of 318 athletes from the 2013-2023 NBA first-round draft classes were included in this study; 87 (27.4%) who were identified as multisport athletes in high school and 231 (72.6%) single-sport athletes. Of the multisport cohort, 76 (87.4%) players participated in high school sports in the United States, while 11 (12.6%) players were in foreign countries, and of the single-sport players, 193 (83.5%) participated in high school basketball domestically and 38 (16.5%) were in other countries. When analyzing the total number of injuries that occurred in our study population according to anatomic location, ankle injury had the highest frequency in both the single-sport (n = 230) and the multisport (n = 81) cohort, followed by knee injury (single-sport: n = 146; multisport: n = 51) (Table 1). The total number of injuries that occurred in the single-sport and multisport athletes was reflective of their cohort sizes (72.6% and 27.4%, respectively).

**Table 1:** Injuries According to Anatomic Location in the Single-Sport and Multisport Cohorts

Injury Location	Single-Sport	Multisport	Overall
	(n = 231 Athletes; 72.6%)	(n = 87 Athletes; 27.4%)	(n = 318 Athletes)
Abdominal	10	5	15
Achilles	5	3	8
Ankle	230	81	311
Back	55	25	80
Calf	18	5	23
Chest	5	1	6
Foot	91	27	118
Groin	23	5	28
Hamstring	17	6	23
Hip	36	24	60
Knee	146	51	197
Leg (unspecified)	7	11	18
Neck	5	1	6
Other	4	2	6
Quadriceps	27	9	36
Shoulder	41	14	55
Tibia	2	2	4
			
Total injuries	722	272	994

Compared with single-sport athletes, multisport athletes played in significantly more games in their first NBA season (50.8 ± 25.0 vs 41.9 ± 25.2; *P* < .01) and second season (58.6 ± 21.5 vs 50.4 ± 24.4; *P* < .01) (Table 2). During their first season, multisport players also had significantly greater total distance traveled (51,542.3 ± 87,634.3 m vs 22,632.8 ± 55,230.9 m; *P* < .001) (Table 2) and PER (12.8 ± 11.6 vs 10.5 ± 5.1; *P* < .05) compared with single-sport players. Overall, across all 3 seasons, the multisport cohort had significantly more games played (148.9 ± 67.1 vs 125.8 ± 63.8; *P* < .01) and distance traveled (133,183.9 ± 239,923.0 m vs 73,879.5 ± 165,093.9 m; *P* < .01) than the single-sport cohort. There were no significant differences in the number of injuries or major injuries between single-sport and multisport athletes within individual seasons or overall (overall number of injuries: 3.1 ± 2.7 vs 3.1 ± 2.4; *P* = .99; the overall number of major injuries: 0.6 ± 0.8 vs 0.6 ± 0.8; *P* = .86) (Table 2).

**Table 2:** Comparison of Workload, Injury, and Statistical Success Between Single-Sport and Multisport Players^*a*^

Players	Games Played, n	Total Distance Traveled, m	Distance per Game (m per game)	Injuries, n	Major Injuries, n^*b*^	PER
First season						
Single-Sport	41.9 ± 25.2	22,632.8 ± 55,230.9	505.5 ± 1520.6	1.0 ± 1.2	0.2 ± 0.4	10.5 ± 5.1
Multisport	50.8 ± 25.0	51,542.3 ± 87,634.3	784.6 ± 1160.0	1.2 ± 1.4	0.2 ± 0.5	12.8 ± 11.6
*P*	**<.01**	**<.001**	.12	.38	.46	**<.05**
Second season						
Single-Sport	50.4 ± 24.4	27,908.1 ± 69,544.9	454.0 ± 965.3	1.2 ± 1.2	0.2 ± 0.5	12.9 ± 6.6
Multisport	58.6 ± 21.5	45,373.6 ± 25,070.2	630.7 ± 1176.5	1.1 ± 1.1	0.2 ± 0.4	13.1 ± 4.8
*P*	**<.01**	.08	.19	.70	.74	.75
Third season						
Single-Sport	47.9 ± 23.8	32,460.4 ± 71,964.0	534.8 ± 1048.8	1.3 ± 1.4	0.2 ± 0.4	13.2 ± 5.8
Multisport	53.6 ± 26.3	48,195.9 ± 97,646.0	692.7 ± 1328.1	1.1 ± 1.1	0.2 ± 0.4	13.2 ± 6.9
*P*	.09	.16	.31	.38	.52	.99
Overall						
Single-Sport	125.8 ± 63.8	73,879.5 ± 165,093.9	471.1 ± 916.9	3.1 ± 2.7	0.6 ± 0.8	11.5 ± 3.8
Multisport	148.9 ± 67.1	133,183.9 ± 239,923.0	680.1 ± 1093.8	3.1 ± 2.4	0.6 ± 0.8	12.3 ± 3.2
*P*	**<.01**	**<.05**	.09	.99	.86	.09

a Data are reported as mean ± SD. Boldface *P* values indicate statistically significant difference between single-sport and multisport groups (*P* < .05). PER, player efficiency rating.

b Defined as injury that led to the player's missing >10 games.

When accounting for the total number of games played, multisport athletes had a significantly lower percentage of games missed due to injury compared with single-sport athletes (13.5% vs 16.9%; *P* < .001) (Table 3).

**Table 3:** Comparison of Aggregate Games Missed Due to Injury Between Single-Sport and Multisport Players

Players	Games Missed Due to Injury, n	Total Games Played, n	Total Possible Games, n	% Games Missed Due to Injury
Single-Sport	5911	29,050	34,961	16.9^*a*^
Multisport	2030	12,957	14,987	13.5^*a*^
Total	7941	42,007	49,948	15.9

a Significant difference between groups (*P* < .001).

Additionally, there were significant correlations between workload and the number of injuries within the single-sport cohort that were not seen in the multisport athletes. Single-sport athletes had significant correlations between increased number of games played and increased number of injuries (*ρ* = 0.32; *P* < .001) (Table 4). There were also positive correlations in their total distance traveled with the number of injuries (*ρ* = 0.37; *P* < .001) and major injuries (*ρ* = 0.18; *P* < .01). For multisport players, no significant correlations were found between any workload category and number of injuries (Table 4). When combining all athletes, there was a similar profile of significant correlations that were also seen for the single-sport cohort, such as between games played and number of injuries (*ρ* = 0.23; *P* < .001) (Table 4).

**Table 4:** Correlations Between Workload and Injuries in Single-Sport and Multisport Players

Players	Number of Injuries		Number of Major Injuries^*a*^	
	*ρ*	*P*	*ρ*	*P*
Single-Sport				
Games played	0.32	<.001	0.01	.90
Total distance traveled, m	0.37	<.001	0.18	<.01
Distance per game, m per game	0.00	.99	0.09	.18
Multisport				
Games played	−0.03	.80	−0.19	.08
Total distance traveled, m	0.14	.20	0.07	.55
Distance per game, m per game	−0.07	.54	0.01	.94
Total				
Games played	0.23	<.001	−0.05	.42
Total distance traveled, m	0.31	<.001	0.14	<.05
Distance per game, m per game	−0.01	.85	0.07	.24

a Defined as injury that led to the player's missing >10 games.

Last, multisport athletes were found to have been significantly more likely to achieve end-of-season awards than single-sport athletes (40.2% vs 19.0%; *P* < .001) (Table 5).

**Table 5:** Comparison of Performance Award Success Between Single-Sport and Multisport Players^*a*^

Players	Achieved ≥1 Award, n	Did Not Achieve an Award, n	% Players Who Achieved an Award
Single-Sport	44	187	19.0^*b*^
Multisport	35	52	40.2^*b*^
Total	79	239	24.8

a End-of-season award achievement was defined by a player's being selected to an All-Rookie or All-NBA team at least once in his first 3 seasons after being drafted.

b Significant difference between groups (*P* < .001).

## Discussion

In this study, we evaluated the differences between elite athletes in their ability to withstand higher workloads and subsequent injury risk based on their sport specialization status as high school athletes. Previous studies analyzing the effects of sport specialization have been limited by small sample sizes, a focus on Olympic sports, or a lack of comparison among athletes’ ability to tolerate different amounts of physical load at the professional level.^13,30,42^ Findings indicated that elite NBA athletes who were multisport athletes in high school were able to handle significantly higher game and season workloads, as measured by the distance traveled and games played, than those who were single-sport athletes. Further, these multisport players had fewer games missed due to injury compared with their single-sport peers. Also, positive correlations between workload and injury that were seen in the single-sport and the combined cohort were absent in the multisport cohort. Finally, results indicated that NBA players with multisport backgrounds had significantly greater statistical and award success in their professional careers than single-sport athletes.

The misconception that early sport specialization creates an advantage in professional athletic success has been shown to be largely untrue in individual sports such as swimming and tennis.^7,11^ Those who participate in multiple sports as a youth athlete are more likely to achieve elite status in their respective field later on.^14,25^ In our study, we showed that among elite-level basketball players, playing multiple sports during high school was associated with significantly higher PER and a greater likelihood of end-of-season award achievement compared with single-sport athletes. These findings suggest that elite team sport athletes see the same benefit in their professional career from playing multiple sports in their youth as previously found in individual sport athletes. This could be attributed to these athletes having developed improved physical traits such as gross motor coordination and overall strength from more diverse sporting experiences.^12^ It is possible, however, that athletes with improved physical traits were also more likely to excel and compete in more youth sports. Alternatively, due to the increased resilience to workload and lower injury rates, multisport athletes might have been able to participate in more practices and training sessions for additional skill and game-knowledge development at the professional level.

Recently, the effects of workload on athletes and their injury outcomes have also become a topic of interest, particularly within the NBA. The concept of load management was created by NBA teams and media to describe the practice of strategically resting players in hopes of minimizing physiologic workload and preventing injury.^21,32^ Previous studies in elite team sport athletes have found that injuries were associated with increased physical workload and fatigue.^1,24,32,36^ However, there are still no studies that investigate the effect of early sport specialization status on athlete resilience to workload. Our findings showed that through their first 3 seasons, NBA players who participated in multiple sports in high school had significantly greater workloads than single-sports players but still had the same number of injuries. Further, while single-sport players had significant correlations between increased workloads and injury, multisport players did not. These findings suggest that playing multiple sports in high school may contribute to future resilience in a professional career to handle a more physical workload without increasing injury risk. This supports previous studies that have suggested that participating in multiple sports as an adolescent could lead to more diverse neuromuscular training and, consequently, protective effects against injury.^8,23,35^ The lack of correlation between workload and the number of injuries in multisport players could also suggest that these athletes are not at greater risk of injury with overuse and may suffer more of their injuries due to other external sporting factors such as incidental player-to-player contact.

One crucial area of emphasis is the increased risk of injury with early sport specialization. We show here that NBA players who only participated in basketball during high school had a significantly greater percentage of games missed due to injury than their multisport peers. Previous studies have shown that elite athletes across multiple different sports who specialized in their respective sport by high school were more likely to have shorter careers and sustain major injury during their careers.^8,35^ Further, these single-sport athletes were more likely to suffer overuse injury due to higher training volumes and exposure hours.^2,20,34^ For example, sport specialization has been shown to put athletes at increased risk of developing chronic anterior knee pain.^15^ Our results support these findings and suggest that single-sport athletes have a higher risk and injury rate than multisport athletes. Thus, the recent rise in injury rates in youth athletes could be partially attributed to the concurrent increase in earlier sport specialization.^17,37,38,44^

### Limitations

This study has several limitations. First, using internet-based information for sport specialization classification could lead to inaccuracies. However, this methodology to gather data has been utilized in multiple other studies analyzing professional athletes in the United States, and this study only included recent first-round draft classes that have seen heavy internet coverage throughout their careers.^8,27,28,35,43^ Second, this study only included elite male basketball players. As the definition of early sport specialization provided by the American Academy of Pediatrics is centered around the onset of puberty, the high school time point selected for sport specialization status may not be as applicable for female youth athletes due to an earlier age of puberty.^3,40^ Additional studies are needed to determine if these findings apply to elite female basketball players and professional athletes in other sports. Third, as detailed injury and health information about athletes is limited to the public, we could not compare workload with different mechanisms of injury or severity of initial diagnosis. It is important to note that our injury data were still consistent with other extensive epidemiologic studies on NBA players that also found that ankle and knee injuries were the most common injuries.^9,35^ Nonetheless, injury studies that utilize the official NBA injury database, which we did not have access to, could provide the most accurate information for this analysis.

Next, this study included first-round picks that were foreign athletes to maximize our sample size. While we attempted to analyze participation in other sports within the same age range as typical US high school athletes, differences in the infrastructure of high school sports or lack thereof could affect the accuracy of adolescent sporting history for these foreign athletes. Additionally, based on previous studies on NBA players that have found no differences in height, weight, position played, or body mass index, between multisport and single-sport athletes,^35^ the analysis in this study between multisport and single-sport cohorts was not separated with these metrics. Our study's utilization of more recent NBA draft classes may have had different characteristics and could have been affected by potential body type factors, such as taller athletes being less likely to participate in other sports or being predisposed to overuse injury due to inherently greater loads. Last, postseason games were excluded from our study due to an effort to standardize player game opportunities, as NBA teams do not all play the same number of postseason contests. Further, postseason games typically require different playstyles and intensity from the regular season, which may contribute confounding factors to workload.

## Conclusion

Basketball players in the NBA, who participate in multiple sports during high school, were found to be more resilient to physical workload and have fewer games missed due to injury in their first 3 seasons, compared with those who specialize in basketball alone. Further, multisport athletes had significantly more athletic success in their professional careers from both a statistical and an award perspective. These findings should be emphasized to youth athletes, parents, and coaches to encourage participation in multiple sports during high school.

## Appendix

**Table A1:** Common Internet Search Patterns for Injury Data Collection

Player-specific search patterns
Player Name + Date +“Injury”
Player Name + Date +“Injury Report”
Player Name + Date + Injury Classification (eg, “Ankle Injury”)
Player Name + Date + Effected Anatomy (eg, “Knee”)
Player Name + Game Number + Season Year +“Injury”
Player Name + Game Number + Season Year +“Injury Report”
Player Name + Game Number + Season Year + Injury Classification
Player Name + Game Number + Season Year + Affected Anatomy
Player Name + Season Year +“Injury”
Player Name + Season Year +“Injuries”
Player Name + Season Year +“Injury Report”
Player Name + Season Year +“Injury History”
Player Name + Season Year + Injury Classification
Player Name + Season Year + Affected Anatomy
Player Name +“Injury”
Player Name +“Injuries”
Player Name +“Injury Report”
Player Name +“Injury History”
Player Name + Injury Classification
Player Name + Affected Anatomy
Team-specific search patterns
Team Name + Date +“Injury”
Team Name + Date +“Injury Report”
Team Name + Game Number + Season Year +“Injury”
Team Name + Game Number + Season Year +“Injury Report”
Team Name + Season Year +“Injury”
Team Name + Season Year +“Injuries”
Team Name + Season Year +“Injury Report”
Team Name + Season Year +“Injury History”
General search patterns
“NBA”+ Date +“Injury”
“NBA”+ Date +“Injury Report”
“NBA”+ Game Number + Season Year +“Injury”
“NBA”+ Game Number + Season Year +“Injury Report”
“NBA”+ Season Year +“Injury”
“NBA”+ Season Year +“Injuries”
“NBA”+ Season Year +“Injury Report”
“NBA”+ Season Year +“Injury History”

## References

[1] Bacon CS Mauger AR. Prediction of overuse injuries in professional U18-U21 footballers using metrics of training distance and intensity. J Strength Cond Res. 2017;31(11):3067-3076. doi:10.1519/JSC.0000000000001744 27930446

[2] Bell DR Post EG Trigsted SM Hetzel S McGuine TA Brooks MA. Prevalence of sport specialization in high school athletics: a 1-year observational study. Am J Sports Med. 2016;44(6):1469-1474. doi:10.1177/0363546516629943 26920433

[3] Brenner JS ; Council on Sports Medicine and Fitness. Sports specialization and intensive training in young athletes. Pediatrics. 2016;138(3):e20162148. doi:10.1542/peds.2016-2148 27573090

[4] Brooks MA Post EG Trigsted SM , et al. Knowledge, attitudes, and beliefs of youth club athletes toward sport specialization and sport participation. Orthop J Sports Med. 2018;6(5):2325967118769836. doi:10.1177/2325967118769836 PMC5946645 29770341

[5] Bruce JR Andrews JR . Ulnar collateral ligament injuries in the throwing athlete. J Am Acad Orthop Surg. 2014;22(5):315-325. doi:10.5435/JAAOS-22-05-315 24788447

[6] Buckley PS Ciccotti MC Bishop M , et al. Youth single-sport specialization in professional baseball players. Orthop J Sports Med. 2020;8(3):2325967120907875. doi:10.1177/2325967120907875 PMC7092410 32232067

[7] Carlson R. The socialization of elite tennis players in Sweden: an analysis of the players’ backgrounds and development. Soc Sport J. 1988;5(3):241-256. doi:10.1123/ssj.5.3.241

[8] Confino J Irvine JN O’Connor M Ahmad CS Lynch TS. Early sports specialization is associated with upper extremity injuries in throwers and fewer games played in Major League Baseball. Orthop J Sports Med. 2019;7(7):2325967119861101. doi:10.1177/2325967119861101 PMC6661792 31384622

[9] Drakos MC Domb B Starkey C Callahan L Allen AA. Injury in the National Basketball Association: a 17-year overview. Sports Health. 2010;2(4):284-290. doi:10.1177/1941738109357303 23015949 PMC3445097

[10] Ericsson KA Th Krampe R Heizmann S. Can we create gifted people? In: Bock GR Ackrill K , eds. Ciba Foundation Symposium 178—The Origins and Development of High Ability. John Wiley & Sons, Ltd. 1993;222-249. doi:10.1002/9780470514498.ch14 8168368

[11] Feeley BT Agel J LaPrade RF. When is it too early for single sport specialization? Am J Sports Med. 2016;44(1):234-241. doi:10.1177/0363546515576899 25825379

[12] Fransen J Pion J Vandendriessche J , et al. Differences in physical fitness and gross motor coordination in boys aged 6-12 years specializing in one versus sampling more than one sport. J Sports Sci. 2012;30(4):379-386. doi:10.1080/02640414.2011.642808 22214429

[13] Güllich A. International medallists’ and non-medallists’ developmental sport activities—a matched-pairs analysis. J Sports Sci. 2017;35(23):2281-2288. doi:10.1080/02640414.2016.1265662 27923322

[14] Güllich A Emrich E. Evaluation of the support of young athletes in the elite sports system. Eur J Sport Society. 2006;3(2):85-108. doi:10.1080/16138171.2006.11687783

[15] Hall R Barber Foss K Hewett TE Myer GD. Sport specialization's association with an increased risk of developing anterior knee pain in adolescent female athletes. J Sport Rehabil. 2015;24(1):31-35. doi:10.1123/jsr.2013-0101 24622506 PMC4247342

[16] Hill GM Simons J. A study of the sport specialization on high school athletics. J Sport Soc Issues. 1989;13(1):1-13. doi:10.1177/019372358901300101

[17] Hodgins JL Vitale M Arons RR Ahmad CS. Epidemiology of medial ulnar collateral ligament reconstruction: a 10-year study in New York State. Am J Sports Med. 2016;44(3):729-734. doi:10.1177/0363546515622407 26797699

[18] Hume PA Hopkins WG Robinson DM Robinson SM Hollings SC. Predictors of attainment in rhythmic sportive gymnastics. J Sports Med Phys Fitness. 1993;33(4):367-377. 8035585

[19] Jayanthi N Pinkham C Dugas L Patrick B Labella C. Sports specialization in young athletes: evidence-based recommendations. Sports Health. 2013;5(3):251-257. doi:10.1177/1941738112464626 24427397 PMC3658407

[20] Jayanthi NA LaBella CR Fischer D Pasulka J Dugas LR. Sports-specialized intensive training and the risk of injury in young athletes: a clinical case-control study. Am J Sports Med. 2015;43(4):794-801. doi:10.1177/0363546514567298 25646361

[21] Jildeh TR. Editorial commentary: load management is essential to prevent season-ending injuries in the National Basketball Association. Arthroscopy. 2024;40(9):2474-2476. doi:10.1016/j.arthro.2024.02.024 38417642

[22] Kliethermes SA Marshall SW LaBella CR , et al. Defining a research agenda for youth sport specialisation in the USA: the AMSSM Youth Early Sport Specialization Summit. Br J Sports Med. 2021;55(3):135-143. doi:10.1136/bjsports-2020-102699 33462103

[23] Ladenhauf HN Graziano J Marx RG. Anterior cruciate ligament prevention strategies: are they effective in young athletes—current concepts and review of literature. Curr Opin Pediatr. 2013;25(1):64. doi:10.1097/MOP.0b013e32835ad208 23274428

[24] Lewis M. It’s a hard-knock life: game load, fatigue, and injury risk in the National Basketball Association. J Athl Train. 2018;53(5):503-509. doi:10.4085/1062-6050-243-17 29771139 PMC6107769

[25] Lidor R Lavyan NZ. A retrospective picture of early sport experiences among elite and near-elite Israeli athletes: developmental and psychological perspectives. Int J Sport Psych. 2002;33:269-289.

[26] Lord M. Too much, too soon? Doctors group warns against early specialization. US News World Rep. 2000;129(3):46. 10977754

[27] Mai HT Alvarez AP Freshman RD , et al. The NFL Orthopaedic Surgery Outcomes Database (NO-SOD): the effect of common orthopaedic procedures on football careers. Am J Sports Med. 2016;44(9):2255-2262. doi:10.1177/0363546516651426 27311414

[28] Mai HT Chun DS Schneider AD , et al. Performance-based outcomes after anterior cruciate ligament reconstruction in professional athletes differ between sports. Am J Sports Med. 2017;45(10):2226-2232. doi:10.1177/0363546517704834 28510477

[29] McLellan M Allahabadi S Pandya NK. Youth sports specialization and its effect on professional, elite, and Olympic athlete performance, career longevity, and injury rates: a systematic review. Orthop J Sports Med. 2022;10(11):23259671221129594. doi:10.1177/23259671221129594 PMC9638532 36353394

[30] Moesch K Elbe AM Hauge MLT Wikman JM. Late specialization: the key to success in centimeters, grams, or seconds (cgs) sports. Scand J Med Sci Sports. 2011;21(6):e282-e290. doi:10.1111/j.1600-0838.2010.01280.x 21401722

[31] Olsen SJ Fleisig GS Dun S Loftice J Andrews JR. Risk factors for shoulder and elbow injuries in adolescent baseball pitchers. Am J Sports Med. 2006;34(6):905-912. doi:10.1177/0363546505284188 16452269

[32] Orringer MJ Pandya NK. Acutely increased workload is correlated with significant injuries among National Basketball Association players. Int J Sports Sci Coach. 2022;17(3):568-575. doi:10.1177/17479541211037037

[33] Post EG Thein-Nissenbaum JM Stiffler MR , et al. High school sport specialization patterns of current Division I athletes. Sports Health. 2017;9(2):148-153. doi:10.1177/1941738116675455 27807260 PMC5349389

[34] Rose MS Emery CA Meeuwisse WH. Sociodemographic predictors of sport injury in adolescents. Med Sci Sports Exerc. 2008;40(3):444. doi:10.1249/MSS.0b013e31815ce61a 18379205

[35] Rugg C Kadoor A Feeley BT Pandya NK. The effects of playing multiple high school sports on National Basketball Association players’ propensity for injury and athletic performance. Am J Sports Med. 2018;46(2):402-408. doi:10.1177/0363546517738736 29135275

[36] Seminati E Minetti AE. Overuse in volleyball training/practice: a review on shoulder and spine-related injuries. Eur J Sport Sci. 2013;13(6):732-743. doi:10.1080/17461391.2013.773090 24251752

[37] Sheu Y Chen LH Hedegaard H. Sports- and recreation-related injury episodes in the United States, 2011-2014. Natl Health Stat Report. 2016;99:1-12. 27906643

[38] Smith NA Chounthirath T Xiang H. Soccer-related injuries treated in emergency departments: 1990–2014. Pediatrics. 2016;138(4):e20160346. doi:10.1542/peds.2016-0346 27621412

[39] Steinl GK Padaki AS Irvine JN Popkin CA Ahmad CS Lynch TS. The prevalence of high school multi-sport participation in elite national football league athletes. Phys Sportsmed. 2021;49(4):476-479. doi:10.1080/00913847.2020.1856632 33238784

[40] Sun SS Schubert CM Chumlea WC , et al. National estimates of the timing of sexual maturation and racial differences among US children. Pediatrics. 2002;110(5):911-919. doi:10.1542/peds.110.5.911 12415029

[41] The National Federation of State High School Associations. 2018-19 high school athletics participation survey. Accessed May 4, 2024. https://www.nfhs.org/media/1020412/2018-19_participation_survey.pdf

[42] Vaeyens R Güllich A Warr CR Philippaerts R. Talent identification and promotion programmes of Olympic athletes. J Sports Sci. 2009;27(13):1367-1380. doi:10.1080/02640410903110974 19787538

[43] Watkins RG Hanna R Chang D Watkins RG. Return-to-play outcomes after microscopic lumbar diskectomy in professional athletes. Am J Sports Med. 2012;40(11):2530-2535. doi:10.1177/0363546512458570 22986297

[44] Zhang AL Sing DC Rugg CM Feeley BT Senter C. The rise of concussions in the adolescent population. Orthop J Sports Med. 2016;4(8):2325967116662458. doi:10.1177/2325967116662458 PMC4989377 27579334

